# Supplementary material for: Factors associated with the occurrence and persistence of subthreshold and full attention-deficit hyperactivity disorder in women: A population-based epidemiological study
Source: PLoS One. 2026 May 14;21(5):e0340179. doi: 10.1371/journal.pone.0340179 (PMC13175469; doi:10.1371/journal.pone.0340179)
Supplement: S1 File — S2 Text: Psychiatric, psychological and somatic assessments. S3 Text: Theoretical and methodological considerations in LCA/ LPA on complex targets. S4 Table: Retrospectively reported childhood ADHD symptoms in women. S5 Table: Raw values of marker variables by measurement, overall sample, women. S6 Table: Subthreshold ADHD in women: model fit indices in LCA/ LPA, classes 1–4. S7 Table: Full ADHD in women: model fit indices in LCA/ LPA, classes 1–3. S8 Text: References. S9 Table: Low-level aggregate data (examples). (ZIP) [file pone.0340179.s001.zip › S9_table.pdf]

S9a: Low-level aggregate data (example): subthreshold ADHD vs. selected variables

subthreshold ADHD	inter-parental violence	fear of parental maltreatment	N Obs	Variable	N	N Miss	Mean	Std Error
0.00	0.00	0.00	2063	systolic BP	2062	1	0.014	0.017
				diastolic BP	2061	2	0.016	0.018
				PBI care mother	1136	927	25.371	0.253
				PBI care father	1099	964	23.898	0.257
				PBI autonomy denial mother	1136	927	5.954	0.129
				PBI autonomy denial father	1099	964	4.280	0.109
		1.00	135	systolic BP	135	0	-0.089	0.065
				diastolic BP	135	0	-0.113	0.067
				PBI care mother	90	45	15.949	1.084
				PBI care father	82	53	16.519	1.116
				PBI autonomy denial mother	90	45	8.129	0.597
				PBI autonomy denial father	82	53	5.354	0.428
	1.00	0.00	190	systolic BP	190	0	0.041	0.050
				diastolic BP	190	0	0.050	0.057
				PBI care mother	116	74	21.688	0.790
				PBI care father	113	77	16.602	0.771
				PBI autonomy denial mother	116	74	6.922	0.428
				PBI autonomy denial father	113	77	6.119	0.387
		1.00	109	systolic BP	109	0	-0.074	0.074
				diastolic BP	109	0	-0.097	0.080
				PBI care mother	75	34	17.408	1.223
				PBI care father	70	39	14.998	1.032
				PBI autonomy denial mother	75	34	7.467	0.541
				PBI autonomy denial father	70	39	6.951	0.513
1.00	0.00	0.00	49	systolic BP	49	0	-0.142	0.125
				diastolic BP	49	0	-0.213	0.139
				PBI care mother	28	21	23.321	1.909
				PBI care father	28	21	22.200	1.888
				PBI autonomy denial mother	28	21	7.114	0.822
				PBI autonomy denial father	28	21	6.149	0.715
		1.00	8	systolic BP	8	0	-0.373	0.369
				diastolic BP	8	0	0.124	0.457
				PBI care mother	5	3	17.200	4.727
				PBI care father	4	4	19.000	3.536
				PBI autonomy denial mother	5	3	9.200	2.672

			PBI autonomy denial father	4	4	8.250	2.594
1.00	0.00	16	systolic BP	16	0	−0.167	0.215
			diastolic BP	16	0	−0.332	0.254
			PBI care mother	9	7	17.212	3.107
			PBI care father	9	7	14.619	2.024
			PBI autonomy denial mother	9	7	9.444	1.375
			PBI autonomy denial father	9	7	8.111	1.791
	1.00	9	systolic BP	9	0	−0.078	0.276
			diastolic BP	9	0	−0.326	0.395
			PBI care mother	7	2	20.286	2.598
			PBI care father	5	4	15.800	5.894
			PBI autonomy denial mother	7	2	9.286	2.407
			PBI autonomy denial father	5	4	3.000	1.414

S9b: Low-level aggregate data (example): subthreshold ADHD vs. selected variables

persistent subthreshold ADHD	inter-parental violence	fear of parental maltreatment	N Obs	Variable	N	N Miss	Mean	Std Error
0.00	0.00	0.00	32	systolic BP	32	0	-0.290	0.164
				diastolic BP	32	0	-0.423	0.176
		1.00	3	systolic BP	3	0	-0.955	0.479
				diastolic BP	3	0	-0.554	0.443
	1.00	0.00	5	systolic BP	5	0	-0.583	0.354
				diastolic BP	5	0	-0.848	0.491
		1.00	6	systolic BP	6	0	-0.260	0.402
				diastolic BP	6	0	-0.725	0.472
1.00	0.00	0.00	17	systolic BP	17	0	0.135	0.175
				diastolic BP	17	0	0.181	0.199
		1.00	5	systolic BP	5	0	-0.024	0.480
				diastolic BP	5	0	0.531	0.645
	1.00	0.00	11	systolic BP	11	0	0.021	0.258
				diastolic BP	11	0	-0.097	0.281
		1.00	3	systolic BP	3	0	0.285	0.079
				diastolic BP	3	0	0.471	0.531

S9c: Low-level aggregate data (example): subthreshold ADHD vs. selected variables

full ADHD	inter-parental violence	fear of parental maltreatment	N Obs	Variable	N	N Miss	Mean	Std Error
0	0.00	0.00	2132	PBI care mother	1177	955	25.256	0.252
				PBI care father	1139	993	23.855	0.254
				PBI autonomy denial mother	1177	955	5.996	0.127
				PBI autonomy denial father	1139	993	4.341	0.107
		1.00	146	PBI care mother	97	49	15.943	1.031
				PBI care father	88	58	16.665	1.068
				PBI autonomy denial mother	97	49	8.202	0.568
				PBI autonomy denial father	88	58	5.523	0.418
	1.00	0.00	209	PBI care mother	127	82	21.407	0.763
				PBI care father	125	84	16.732	0.730
				PBI autonomy denial mother	127	82	7.110	0.406
				PBI autonomy denial father	125	84	6.348	0.379
		1.00	118	PBI care mother	82	36	17.654	1.140
				PBI care father	75	43	15.052	1.026
				PBI autonomy denial mother	82	36	7.622	0.534
				PBI autonomy denial father	75	43	6.688	0.499
1	0.00	0.00	21	PBI care mother	15	6	20.200	2.156
				PBI care father	12	9	18.859	2.612
				PBI autonomy denial mother	15	6	9.600	1.241
				PBI autonomy denial father	12	9	5.333	1.534
		1.00	3	PBI care mother	2	1	19.956	8.956
				PBI care father	2	1	25.000	0.000
				PBI autonomy denial mother	2	1	10.500	4.500
				PBI autonomy denial father	2	1	11.500	4.500
	1.00	0.00	8	PBI care mother	5	3	17.800	3.625
				PBI care father	5	3	16.400	4.589
				PBI autonomy denial mother	5	3	11.600	1.030
				PBI autonomy denial father	5	3	8.000	1.897
		1.00	9	PBI care mother	7	2	8.428	2.741
				PBI care father	6	3	12.167	4.989
				PBI autonomy denial mother	7	2	7.199	1.392
				PBI autonomy denial father	6	3	4.957	1.587

S9d: Low-level aggregate data (example): subthreshold ADHD vs. selected variables

persistent full ADHD	inter-parental violence	fear of parental maltreatment	N Obs	Variable	N	N Miss	Mean	Std Error
0.00	0.00	0.00	11	monocytes	10	1	−0.681	0.308
				neutrophils	10	1	−0.365	0.314
				hsCRP	11	0	−0.245	0.262
				insulin	11	0	−0.272	0.194
				leptin	11	0	−0.335	0.160
		1.00	2	monocytes	1	1	−0.000	.
				neutrophils	1	1	0.068	.
				hsCRP	2	0	−0.708	0.095
				insulin	2	0	−0.877	0.222
				leptin	2	0	−0.980	0.502
	1.00	0.00	3	monocytes	2	1	−0.108	0.477
				neutrophils	2	1	−0.838	0.051
				hsCRP	3	0	−0.147	0.728
				insulin	3	0	0.018	0.515
				leptin	3	0	−0.629	0.566
		1.00	2	monocytes	1	1	−0.077	.
				neutrophils	1	1	−1.478	.
				hsCRP	2	0	0.641	0.208
				insulin	2	0	−0.344	0.131
				leptin	2	0	−0.643	0.796
1.00	0.00	0.00	10	monocytes	9	1	0.141	0.181
				neutrophils	9	1	0.014	0.384
				hsCRP	10	0	0.451	0.278
				insulin	10	0	−0.007	0.210
				leptin	10	0	0.229	0.351
		1.00	1	monocytes	*			
				neutrophils	*			
				hsCRP	*			
				insulin	*			
				leptin	*			
	1.00	0.00	5	monocytes	4	1	0.416	0.525
				neutrophils	4	1	0.373	0.771
				hsCRP	5	0	0.592	0.256
				insulin	5	0	0.165	0.329

		leptin	5	0	0.180	0.502
1.00	7	monocytes	4	3	0.522	0.383
		neutrophils	4	3	1.438	0.866
		hsCRP	7	0	0.067	0.350
		insulin	6	1	0.444	0.423
		leptin	7	0	0.134	0.300

* individual data cannot be shown due to limitations included in confirmed consent
