# Supplementary material for: Factors associated with the occurrence and persistence of subthreshold and full attention-deficit hyperactivity disorder in women: A population-based epidemiological study
Source: PLoS One. 2026 May 14;21(5):e0340179. doi: 10.1371/journal.pone.0340179 (PMC13175469; doi:10.1371/journal.pone.0340179)
Supplement: S1 File — S2 Text: Psychiatric, psychological and somatic assessments. S3 Text: Theoretical and methodological considerations in LCA/ LPA on complex targets. S4 Table: Retrospectively reported childhood ADHD symptoms in women. S5 Table: Raw values of marker variables by measurement, overall sample, women. S6 Table: Subthreshold ADHD in women: model fit indices in LCA/ LPA, classes 1–4. S7 Table: Full ADHD in women: model fit indices in LCA/ LPA, classes 1–3. S8 Text: References. S9 Table: Low-level aggregate data (examples). (ZIP) [file pone.0340179.s001.zip › S7_table.pdf]

S7: Full ADHD in women: model fit indices in LCA / LPA, classes 1-3

n and fit statistics	1-class	2-class	3-class
n	41	26 / 15	15 / 24 / 2
AIC	277.2	265.0	269.4
BIC	287.5	285.6	300.3
ABIC	268.7	248.0	243.9
bootstrapped LRT p-value	-	.000	.250

Notes:
AIC: Akaike information criterion; BIC: Bayesian information criterion; ABIC: sample-size adjusted BIC; LRT: likelihood ratio test
