# Supplementary material for: Factors associated with the occurrence and persistence of subthreshold and full attention-deficit hyperactivity disorder in women: A population-based epidemiological study
Source: PLoS One. 2026 May 14;21(5):e0340179. doi: 10.1371/journal.pone.0340179 (PMC13175469; doi:10.1371/journal.pone.0340179)
Supplement: S1 File — S2 Text: Psychiatric, psychological and somatic assessments. S3 Text: Theoretical and methodological considerations in LCA/ LPA on complex targets. S4 Table: Retrospectively reported childhood ADHD symptoms in women. S5 Table: Raw values of marker variables by measurement, overall sample, women. S6 Table: Subthreshold ADHD in women: model fit indices in LCA/ LPA, classes 1–4. S7 Table: Full ADHD in women: model fit indices in LCA/ LPA, classes 1–3. S8 Text: References. S9 Table: Low-level aggregate data (examples). (ZIP) [file pone.0340179.s001.zip › S8_text.pdf]

S8 References

1. Firmann M, Mayor V, Vidal PM, Bochud M, Pecoud A, Hayoz D, et al. The CoLaus study: a population-based study to investigate the epidemiology and genetic determinants of cardiovascular risk factors and metabolic syndrome. BMC Cardiovascular Disorders 2008;8:6.
2. Preisig M, Waeber G, Vollenweider P, Bovet P, Rothen S, Vandeleur C, et al. The PsyCoLaus study: methodology and characteristics of the sample of a population-based survey on psychiatric disorders and their association with genetic and cardiovascular risk factors. BMC Psychiatry 2009;9:9.
3. Mohr S, Preisig M, Fenton BT, Ferrero F. Validation of the French version of the Parental Bonding Instrument in adults Personality Individual Differences 1999;26:1065-74.
4. Preisig M, Fenton BT, Matthey ML, Berney A, Ferrero F. Diagnostic interview for genetic studies (DIGS): inter-rater and test-retest reliability of the French version. Eur Arch Psychiatry Clin Neurosci 1999;249(4):174-9.
5. Nurnberger JI, Jr., Blehar MC, Kaufmann CA, York-Cooler C, Simpson SG, Harkavy-Friedman J, et al. Diagnostic interview for genetic studies. Rationale, unique features, and training. NIMH Genetics Initiative. Arch Gen Psychiatry 1994;51(11):849-59.
6. Berney A, Preisig M, Matthey ML, Ferrero F, Fenton BT. Diagnostic interview for genetic studies (DIGS): inter-rater and test-retest reliability of alcohol and drug diagnoses. Drug and Alcohol Dependence 2002;65(2):149-58.
7. Leboyer M, Maier W, Teherani M, Lichtermann D, D'Amato T, Franke P, et al. The reliability of the SADS-LA in a family study setting. Eur Arch Psychiatry Clin Neurosci 1991;241(3):165-9.
8. Endicott J, Spitzer RL. A diagnostic interview: the schedule for affective disorders and schizophrenia. Arch Gen Psychiatry 1978;35(7):837-44.
9. Rougemont-Buecking A, Rothen S, Jeanpretre N, Lustenberger Y, Vandeleur CL, Ferrero F, et al. Inter-informant agreement on diagnoses and prevalence estimates of anxiety disorders: direct interview versus family history method. Psychiatry Res 2008;157(1-3):211-23.
10. Orvaschel H, Puig-Antich J, Chambers W, Tabrizi MA, Johnson R. Retrospective assessment of prepubertal major depression with the Kiddie-SADS-e. J Am Acad Child Psychiatry 1982;21(4):392-7.

11. Merikangas KR, Dierker LC, Szatmari P. Psychopathology among offspring of parents with substance abuse and/or anxiety disorders: a high-risk study. J Child Psychol Psychiatry 1998;39(5):711-20.
12. Merikangas KR, Stolar M, Stevens DE, Goulet J, Preisig MA, Fenton B, et al. Familial transmission of substance use disorders. Arch Gen Psychiatry 1998;55(11):973-9.
13. Perrin M, Vandeleur CL, Castelao E, Rothen S, Glaus J, Vollenweider P, et al. Determinants of the development of post-traumatic stress disorder, in the general population. Soc Psychiatry Psychiatr Epidemiol 2014;49(3):447-57.
14. Parker G, Tupling H, Brown LB. A parental bonding instrument. British Journal of Medical Psychology 1979;52:1-10.
15. Andreasen NC, Endicott J, Spitzer RL, Winokur G. The family history method using diagnostic criteria. Reliability and validity. Arch Gen Psychiatry 1977;34:1229–35.
16. Vandeleur CL, Rothen S, Jeanpretre N, Lustenberger Y, Gamma F, Ayer E, et al. Inter-informant agreement and prevalence estimates for substance use disorders: direct interview versus family history method. Drug and Alcohol Dependence 2008;92(1-3):9-19.
17. Vandeleur CL, Rothen S, Lustenberger Y, Glaus J, Castelao E, Preisig M. Inter-informant agreement and prevalence estimates for mood syndromes: direct interview vs. family history method. Journal of Affective Disorders 2015;171:120-7.
18. Rothen S, Vandeleur CL, Lustenberger Y, Jeanpretre N, Ayer E, Fornerod D, et al. Personality traits in children of parents with unipolar and bipolar mood disorders. Journal of Affective Disorders 2009;113(1-2):133-41.
19. Marques-Vidal P, Bochud M, Bastardot F, Luscher T, Ferrero F, Gaspoz JM, et al. Levels and determinants of inflammatory biomarkers in a Swiss population-based sample (CoLaus study). PLoS One 2011;6(6):e21002.
20. Ajdacic-Gross V, Ajdacic L, Xu Y, Müller M, Rodgers S, Wyss C, et al. Backtracing persistent biomarker shifts to the age of onset: A novel procedure applied to men's and women's white blood cell counts in post-traumatic stress disorder. Biomarkers in Neuropsychiatry 2021;4:1-10.
21. Headache Classification Subcommittee of the International Headache Society. The International Classification of Headache Disorders: 2nd edition. Cephalalgia 2004;24:9–160.
