# Supplementary material for: Factors associated with the occurrence and persistence of subthreshold and full attention-deficit hyperactivity disorder in women: A population-based epidemiological study
Source: PLoS One. 2026 May 14;21(5):e0340179. doi: 10.1371/journal.pone.0340179 (PMC13175469; doi:10.1371/journal.pone.0340179)
Supplement: S1 File — S2 Text: Psychiatric, psychological and somatic assessments. S3 Text: Theoretical and methodological considerations in LCA/ LPA on complex targets. S4 Table: Retrospectively reported childhood ADHD symptoms in women. S5 Table: Raw values of marker variables by measurement, overall sample, women. S6 Table: Subthreshold ADHD in women: model fit indices in LCA/ LPA, classes 1–4. S7 Table: Full ADHD in women: model fit indices in LCA/ LPA, classes 1–3. S8 Text: References. S9 Table: Low-level aggregate data (examples). (ZIP) [file pone.0340179.s001.zip › S6_table.pdf]

S6: Subthreshold ADHD in women: model fit indices in LCA / LPA, classes 1-4

n and fit statistics	1-class	2-class	3-class	4-class
n	81	69 / 12	30 / 40 / 11	30 / 39 / 11 / 1
AIC	1121.3	1081.7	1064.7	1060.3
BIC	1150.1	1134.4	1141.3	1160.9
ABIC	1112.2	1065.0	1040.4	1028.4
bootstrapped LRT p-value		0.000	0.000	0.078

Notes:
AIC: Akaike information criterion; BIC: Bayesian information criterion; ABIC: sample-size adjusted BIC; LRT: likelihood ratio test
