# Supplementary material for: Factors associated with the occurrence and persistence of subthreshold and full attention-deficit hyperactivity disorder in women: A population-based epidemiological study
Source: PLoS One. 2026 May 14;21(5):e0340179. doi: 10.1371/journal.pone.0340179 (PMC13175469; doi:10.1371/journal.pone.0340179)
Supplement: S1 File — S2 Text: Psychiatric, psychological and somatic assessments. S3 Text: Theoretical and methodological considerations in LCA/ LPA on complex targets. S4 Table: Retrospectively reported childhood ADHD symptoms in women. S5 Table: Raw values of marker variables by measurement, overall sample, women. S6 Table: Subthreshold ADHD in women: model fit indices in LCA/ LPA, classes 1–4. S7 Table: Full ADHD in women: model fit indices in LCA/ LPA, classes 1–3. S8 Text: References. S9 Table: Low-level aggregate data (examples). (ZIP) [file pone.0340179.s001.zip › S5_table.pdf]

S5: Raw values of marker variables by measurement, overall sample, women

	baseline measurement			1st follow-up measurement			2nd follow-up measurement			units	data transformation [1]
	N	mean	SE	N	mean	SE	N	mean	SE		
leukocytes	-	-	-	1668	6.180	0.0504	2219	6.101	0.0477	G/l	ln
basophils	-	-	-	1667	0.036	0.0005	2179	0.047	0.0007	G/l	sq
eosinophils	-	-	-	1667	0.166	0.0027	2178	0.164	0.0026	G/l	sq
lymphocytes	-	-	-	1665	1.983	0.0145	2178	1.876	0.0270	G/l	sq
monocytes	-	-	-	1665	0.497	0.0039	2178	0.491	0.0037	G/l	sq
neutrophils	-	-	-	1665	3.443	0.0332	2178	3.496	0.0293	G/l	sq
IL-6	2598	7.997	1.0969	2228	18.11	1.6578	1132	0.649	0.0518	pg/ml	ln
IL-1β	2598	4.108	0.5718	2229	4.351	0.4673	959	0.049	0.0028	pg/ml	ln
TNF-α	2598	7.430	1.4369	2230	10.68	1.7310	1473	1.962	0.1009	pg/ml	ln
hsCRP	2600	1.899	0.0383	2276	1.986	0.0408	2028	1.825	0.0419	ng/ml	ln
BMI	2738	24.93	0.0918	2396	25.51	0.1022	2216	25.82	0.1096	BMI	ln
waist-hip ratio	2737	0.823	0.0013	2410	0.881	0.0013	2219	0.842	0.0016	ratio	-
systolic blood pressure [2]	2735	124.7	0.3608	2411	124.0	0.4056	2224	126.4	0.4279	mmHg	ln
diastolic blood pressure [2]	2735	77.10	0.2015	2411	76.30	0.2180	2224	75.86	0.2188	mmHg	
cholesterol total	2736	5.555	0.0193	2411	5.789	0.0207	2224	5.486	0.0203	mmol/l	-
HDL cholesterol	2736	1.803	0.0079	2411	1.813	0.0093	2224	1.769	0.0097	mmol/l	sq
LDL cholesterol	2728	3.232	0.0173	2401	3.439	0.0187	2214	3.176	0.0186	mmol/l	sq
triglycerides	2736	1.135	0.0125	2411	1.180	0.0135	2224	1.193	0.0192	mmol/l	ln
insulin	2175	7.848	0.1182	2403	7.577	0.1291	2216	9.274	0.2825	microIU/mL	ln
glucose	2736	5.314	0.0189	2411	5.659	0.0189	2223	5.270	0.0203	mmol/l	ln
HBA1c	-	-	-	-	-	-	2221	37.75	0.1193	mmol/mol	-
adiponectin	2486	12204.7	180.14	2230	6099.9	100.16	-	-	-	ng/ml	ln
leptin	2365	16.60	0.2379	2230	6979.5	156.49	-	-	-	ng/ml	sq
salivary cortisol										nmol/l	
awakening response	-	-	-	1145	9.098	0.3642	1405	14.12	0.4720		
AUC / ground	-	-	-	1105	140.1	1.6655	1375	200.7	2.4283		
AUC / increase	-	-	-	1105	-103.4	3.4970	1375	-140.2	4.9259		
diurnal cortisol slope	-	-	-	1156	1.137	0.0221	1393	1.585	0.0310		

PBI mother			
care	1532	23.71	0.2395
denial of autonomy	1532	6.416	0.1165
encouragement of freedom	1532	9.188	0.1120
PBI father			
care	1469	22.17	0.2407
denial of autonomy	1469	4.751	0.1001
encouragement of freedom	1469	9.309	0.1174

Notes:
1. Similarly applied to values from all three measurements.
2. Average of readings 2 and 3.
