# Supplementary material for: Factors associated with the occurrence and persistence of subthreshold and full attention-deficit hyperactivity disorder in women: A population-based epidemiological study
Source: PLoS One. 2026 May 14;21(5):e0340179. doi: 10.1371/journal.pone.0340179 (PMC13175469; doi:10.1371/journal.pone.0340179)
Supplement: S1 File — S2 Text: Psychiatric, psychological and somatic assessments. S3 Text: Theoretical and methodological considerations in LCA/ LPA on complex targets. S4 Table: Retrospectively reported childhood ADHD symptoms in women. S5 Table: Raw values of marker variables by measurement, overall sample, women. S6 Table: Subthreshold ADHD in women: model fit indices in LCA/ LPA, classes 1–4. S7 Table: Full ADHD in women: model fit indices in LCA/ LPA, classes 1–3. S8 Text: References. S9 Table: Low-level aggregate data (examples). (ZIP) [file pone.0340179.s001.zip › S4_table.pdf]

S4: Retrospectively reported childhood ADHD symptoms in women

symptom	DSM-IV criteria	frequency all	rate (%) all	frequency Sx pure	rate (%) Sx pure	frequency Dx pure	rate (%) Dx pure
probe question 1: concentration problems	probe 1	135	5.93	99	3.74	36	1.39
probe question 2: acting without contemplating, fidgets	probe 2	70	2.55	42	1.59	28	1.08
difficulty with details / made careless mistakes	inatt_a	115	4.28	79	2.94	36	1.34
difficulty sustaining attention	inatt_b	80	2.98	48	1.79	32	1.19
did not listen, absent-minded	inatt_c	104	3.87	69	2.57	35	1.30
did not finish tasks	inatt_d1	86	3.20	53	1.97	33	1.23
difficulty organizing tasks and activities	inatt_e	57	2.12	31	1.15	26	0.97
avoided sustained mental efforts	inatt_f	83	3.09	51	1.90	32	1.19
lost things needed for school / work / playing	inatt_g	33	1.23	17	0.63	16	0.60
easily distracted	inatt_h1	54	2.01	29	1.08	25	0.93
forgetful	inatt_i	32	1.19	16	0.60	16	0.60
did not finish tasks, even pleasant ones	inatt_d2	44	1.64	28	1.04	16	0.60
quickly changing desire / focus in activities	inatt_d3	32	1.19	20	0.74	12	0.45
jumping between actions	inatt_h2	45	1.67	27	1.00	18	0.67
restless hands, feet; squirmed in seat	hyper_a	42	1.56	20	0.74	22	0.82
difficulty staying seated	hyper_b	48	1.79	23	0.86	25	0.93
ran around, climbed	hyper_c	26	0.97	13	0.48	13	0.48
difficulty playing or working quietly	hyper_d	28	1.04	12	0.45	16	0.60
felt being propelled, as if driven by a motor	hyper_e1	47	1.75	21	0.78	26	0.97
talked excessively	hyper_f	42	1.56	25	0.93	17	0.63

exposed themselves to danger without thinking	hyper_e2	34	1.27	16	0.60	18	0.67
blurted out answers	impuls_g	31	1.15	14	0.52	17	0.63
difficulty waiting own turn	impuls_h	22	0.82	10	0.37	12	0.45
interrupted / interfered often	impuls_i	21	0.78	5	0.19	16	0.60
