# Supplementary material for: Factors associated with the occurrence and persistence of subthreshold and full attention-deficit hyperactivity disorder in women: A population-based epidemiological study
Source: PLoS One. 2026 May 14;21(5):e0340179. doi: 10.1371/journal.pone.0340179 (PMC13175469; doi:10.1371/journal.pone.0340179)
Supplement: S1 File — S2 Text: Psychiatric, psychological and somatic assessments. S3 Text: Theoretical and methodological considerations in LCA/ LPA on complex targets. S4 Table: Retrospectively reported childhood ADHD symptoms in women. S5 Table: Raw values of marker variables by measurement, overall sample, women. S6 Table: Subthreshold ADHD in women: model fit indices in LCA/ LPA, classes 1–4. S7 Table: Full ADHD in women: model fit indices in LCA/ LPA, classes 1–3. S8 Text: References. S9 Table: Low-level aggregate data (examples). (ZIP) [file pone.0340179.s001.zip › S3_text.pdf]

S3: Theoretical and methodological considerations in LCA / LPA on complex targets

Complexity, complex systems and analyses within this domain on the one hand and latent class analysis on the other hand are both very challenging and controversial topics.

A basic step when analyzing a complex target is to understand and disentangle its inherent heterogeneity. From a system analysis perspective, heterogeneity of a complex disease or disorder like ADHD evolves from different combinations of involved systems / subsystems and their contributions to pathogenetic mechanisms. Heterogeneity is represented by several subtypes of the target disease / disorder with characteristic configurations of involved mechanisms, related risk factors, comorbidity patterns, physiological markers, brain networks etc. A preliminary analysis aim is to disentangle these configurations. On a technical level, these configurations are approachable based on associations patterns and their differentiation along diverse subgroups. The latter can be any manifest subgroups (e.g. sex) or latent subgroups derived from pattern recognition models such as cluster analysis or latent class / latent profile analysis (LCA / LPA – in the following labeled as LCA). Moreover, in a broad sense, almost any analysis can be integrated to contribute to patterns and pattern recognition. Patterns are the clue to disentangle which systems are involved and how they interact to contribute to pathogenetic mechanisms.

Clustering analysis, i.e., subtyping / subgrouping by clusters or classes, is a standard approach in this connection. Clustering is typically accomplished by a cluster analysis or an LCA or by deep clustering in case of big data. Given that there is a variety of conceptions and misconceptions what an LCA can achieve and how it should be implemented, we will clarify in the following to which principles we adhere in analyses of targets featuring a medium or high degree of complexity:

- we consider LCA is a genuine exploratory tool in analysis of complex targets;
- each LCA series within this framework offers different informative solutions both due to enumeration of classes and due to variation in variable selection / definition / aggregation; metaphorically speaking, an LCA series contributes snapshots from different flying altitudes and from different angles; if the target is complex, i.e. a jungle, the snapshots provide many different pictures and impressions;
- any number of classes from two onwards add a new and valuable perspective on a complex target, whereas, in a practical application, the number of classes is limited by technical conditions such as sample size or variable range;
- in congruence with the arguments underlying multiverse regression, the variable selection, definition and aggregation are the dominating sources of uncertainty in LCA;

- all informative solutions which can contribute to the understanding of an analysis need to be assembled and compiled, even though only one or few of them are finally presented to the community;
- we consider interpretability as the priority criterion for understanding and highlighting specific models; adhering to an appropriate uniformity of the matter in variable selection is therefore crucial;
- in contrast to typical statisticians' viewpoints, LCAs within the framework of medium to high degree complexity does not per se offer any optimal solutions; similarly, we do not adhere to ideas such as optimal sample sizes or minimal subgroup sizes; model fit measures, which are often believed to provide an advantage of LCA over other clustering models, are considered as a mere heuristic tool;
- in contrast to conventional thinking revolving around precision, optimization etc., it is the variability of LCA results that mostly contributes to a better understanding of a complex target;
- in the same vein, simple and rough approaches are preferred in analyses with distal variables, instead of hunting for precision, where precision is not targeted.
