# Supplementary material for: Factors associated with the occurrence and persistence of subthreshold and full attention-deficit hyperactivity disorder in women: A population-based epidemiological study
Source: PLoS One. 2026 May 14;21(5):e0340179. doi: 10.1371/journal.pone.0340179 (PMC13175469; doi:10.1371/journal.pone.0340179)
Supplement: S1 File — S2 Text: Psychiatric, psychological and somatic assessments. S3 Text: Theoretical and methodological considerations in LCA/ LPA on complex targets. S4 Table: Retrospectively reported childhood ADHD symptoms in women. S5 Table: Raw values of marker variables by measurement, overall sample, women. S6 Table: Subthreshold ADHD in women: model fit indices in LCA/ LPA, classes 1–4. S7 Table: Full ADHD in women: model fit indices in LCA/ LPA, classes 1–3. S8 Text: References. S9 Table: Low-level aggregate data (examples). (ZIP) [file pone.0340179.s001.zip › S2_text.pdf]

S2: Psychiatric, psychological and somatic assessments

Family-related adverse child events (ACE) were based on answers to the following questions of the DIGS. 1) Interparental violence: "Did your parents fight with each other frequently?" 2) Fear of maltreatment: "Did your parents ever do anything that frightened you (like lock you in a closet)"? These questions, retrospectively documented until the age of 16 years, were taken from a modified version of the Schedule for Affective Disorders and Schizophrenia-Lifetime Version [8] originally used in the Yale Family Study in 1988 [11, 12]. They represent more severe forms of ACE which are proximate to traumatic experiences. The latter were reported in the PTSD section and were limited in the analysis to occurrence below the age of 10, i.e., during childhood. The DIGS section on PTSD retrospectively assessed different types of traumatic events (accidents; physical assaults; witnessing murder, violence or death by accident; sexual abuse; combat and/or war), which were differentially associated with the diagnosis of PTSD in adults, and assessed for their test-retest reliability, except for exposure to war which could not be tested due to its rarity in this sample [13].

Parental bonding was assessed with the "Parental Bonding Instrument" (PBI) [14]. The PBI is a self-report measure of fundamental parental dimensions of parental care and overprotection based on 25 attitudinal and behavioral items (4-point scale) assessed retrospectively for the first 16 years of life. The French version of the PBI revealed three factors: a "parental care" factor as before, and the partitioning of the “overprotection” factor into two separate factors "denial of psychological autonomy" (a negative factor) and "encouragement of behavioral freedom" (a positive factor) [3]. The Cronbach alpha coefficients for these subscales in a community parental sample were 0.86, 0.84 and 0.72, respectively [3]. The three subscales of the PBI were assessed using a reduced subsample of participants. This strategy was typically used to implement additional questionnaires / scales / instruments in an efficient way to the core set of assessment tools.

Information on familial aggregation was assessed using the French version of the semi-structured Family History–Research Diagnostic Criteria (FH-RDC) interview [9, 15-17] and youngsters [18]. Disorders in the FH-RDC were divided into neurodevelopmental disorders (tic disorders, ADHD, conduct disorder, and oppositional defiant disorder), anxiety disorders with a typically early onset, i.e. onset in childhood and early adolescence (separation anxiety, overanxious disorder, specific phobias, social phobia), those with a typically later onset (panic, agoraphobia, generalized anxiety disorder), mood disorders and substance

disorders. Similar to the PBI, the FH-RDC information was only available for a sub-sample of participants.

Data on somatic conditions and biomarkers were taken either from the medical section of the psychiatric interview or from the physical (CoLaus) assessments that had taken place prior to the psychiatric assessments. Morning venous blood samples served to assess inflammatory markers, WBC counts and cardio-metabolic markers. Inflammatory markers comprised IL-1β, IL-6, TNF-α and hsCRP. WBC counts of neutrophils, lymphocytes, monocytes, eosinophils and basophils were computed both as absolute values and as proportions of the total WBC count. Cardio-metabolic markers comprised the BMI based on weight and height, the waist-hip ratio and systolic / diastolic blood pressure all objectively measured by trained nurses, and the levels of glucose, HbA1c, total cholesterol, HDL, LDL, triglycerides, adiponectin and leptin from venous blood samples. The assessment of these biological markers is described in detail elsewhere [1, 19]. The immunological and cardio-metabolic markers were assessed up to three times, i.e., at the baseline examination and at the follow-up 1 and 2 evaluations. The WBC counts were determined only at the follow-ups, adiponectin and leptin at the baseline examination and follow-up 1, and HbA1c only at follow-up 2.

In pre-processing the biomarker data, the Kolmogorov-Smirnov and the Shapiro-Wilk test served to check for the normal distribution of the biomarker variables. If appropriate, the variables were transformed by log or square root. Values were considered as outliers if above / below 3 standard deviations (SD) and were set to missing. In the next step, the variables were z-transformed. This enabled the averaging of the variables if data from two or all three measurements were available. Finally, the variables were age-standardized using a linear or quadratic regression, if appropriate, to exclude bias related to different age patterns of marker variables (referring to the age at measurement) [20].

In addition to the described biological markers, we included comorbid physical diseases in the analysis. Their lifetime history was assessed in the somatic section of the DIGS. The focus was on diseases that typically have an early onset or, alternatively, rely on events or predispositions occurring early in life: infectious childhood diseases, atopic diseases, ulcer, migraine. The lifetime prevalence of migraine was additionally assessed using the French version of the Diagnostic Interview for Headache Syndromes which follows the criteria of the International Classification of Headache Disorders [21].
