# Supplementary material for: Factors associated with the occurrence and persistence of subthreshold and full attention-deficit hyperactivity disorder in women: A population-based epidemiological study
Source: PLoS One. 2026 May 14;21(5):e0340179. doi: 10.1371/journal.pone.0340179 (PMC13175469; doi:10.1371/journal.pone.0340179)

S1: The CoLaus|PsyCoLaus cohort

Data used for the current study were derived from the population-based cohort of CoLaus|PsyCoLaus [1, 2]. The CoLaus|PsyCoLaus study was designed to explore associations between mental disorders and cardiovascular diseases. The cohort was randomly selected from the 35- to 75-year-old residents of the city of Lausanne (Switzerland) from 2003 to 2006 according to the civil register. Figure s1 displays the overall workflow of the sampling process. The initial cohort included 6,734 individuals. The first follow-up (FU1) was carried out from 2009-2013 and the second follow-up (FU2) was conducted from 2014-2018.

At baseline, the psychiatric evaluation, carried out by trained psychologists, was restricted to the 35- to 67-year-old participants in the physical exam, resulting in a 67% participation rate within this age range (N=3,719). From FU1 on, all individuals from the initial cohort were eligible for the psychiatric evaluation. The cohort used in the present paper is comprised of the first assessment of all 5,111 people (35 to 88 years old) who agreed to participate in at least one psychiatric evaluation (N=3,719 at baseline, N=1,155 at FU1, N=237 at FU2).

Instruments

The French version [3, 4] of the semi-structured Diagnostic Interview for Genetic Studies (DIGS) [5], was used to collect diagnostic information on mental disorders. The assessments covered a broad spectrum of the DSM-IV Axis I criteria, as well as the course and chronology of comorbid features. The French version of the DIGS revealed excellent inter-rater reliability in terms of kappa and Yule's Y coefficients for major mood and psychotic disorders [4] as well as for substance use and antisocial personality disorders [6], whereas the 6-week test-retest reliability was slightly lower [3, 6]. The DIGS was completed with the PTSD and the generalized anxiety disorder (GAD) sections of the French version [7] of the Schedule for Affective Disorders and Schizophrenia - Lifetime and Anxiety disorder version (SADS-LA) [8], and the brief phobia chapter of the DIGS was replaced by the corresponding, more extensive chapters of the SADS-LA which elicited detailed information relating to the DSM-IV criteria for agoraphobia with or without panic attacks, social and specific phobias. These anxiety disorders also revealed satisfactory test-retest reliability [7, 9]. ADHD and oppositional defiant disorder were assessed using the corresponding sections of the translated Yale Family Study version of the Schedule for Affective Disorders and Schizophrenia - Lifetime and Anxiety disorder (SADS-LA) [8]. These sections were

developed in analogy to the corresponding sections in the Kiddie-Schedule for Affective Disorders and Schizophrenia - Epidemiologic version (K-SADS-E) [10].

Figure S1: The overall workflow of the CoLaus|PsyCoLaus sampling.

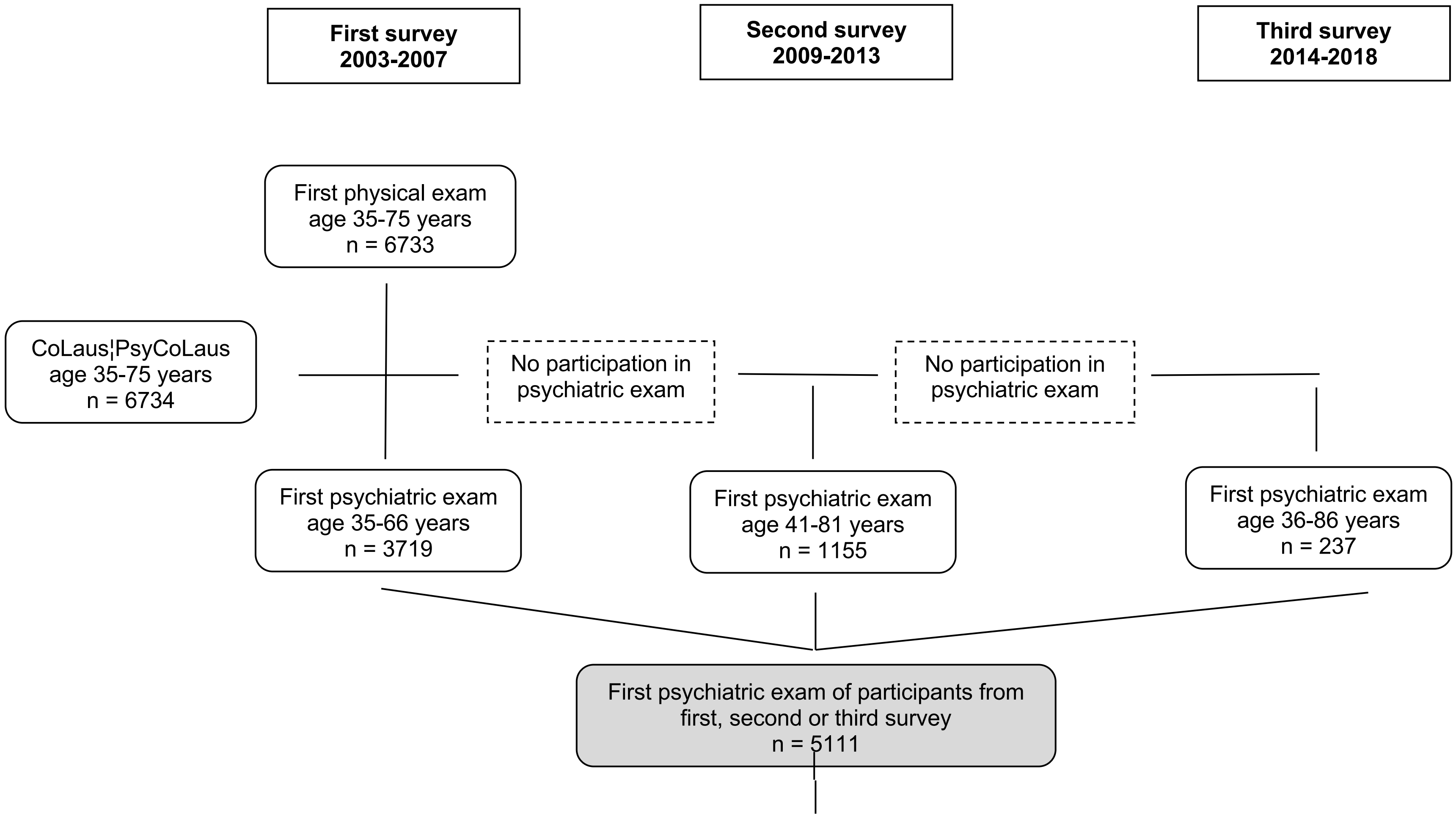

Supplement: S1 File — S2 Text: Psychiatric, psychological and somatic assessments. S3 Text: Theoretical and methodological considerations in LCA/ LPA on complex targets. S4 Table: Retrospectively reported childhood ADHD symptoms in women. S5 Table: Raw values of marker variables by measurement, overall sample, women. S6 Table: Subthreshold ADHD in women: model fit indices in LCA/ LPA, classes 1–4. S7 Table: Full ADHD in women: model fit indices in LCA/ LPA, classes 1–3. S8 Text: References. S9 Table: Low-level aggregate data (examples). (ZIP) [file pone.0340179.s001.zip › S1_text_fig.pdf]
