# Supplementary material for: Factors associated with the occurrence and persistence of subthreshold and full attention-deficit hyperactivity disorder in women: A population-based epidemiological study
Source: PLoS One. 2026 May 14;21(5):e0340179. doi: 10.1371/journal.pone.0340179 (PMC13175469; doi:10.1371/journal.pone.0340179)
Supplement: Attachment — Submitted filename: Response to Reviewers PONE-D-25-65436.docx [file pone.0340179.s002.docx]

PONE-D-25-65436

Factors associated with the occurrence and persistence of subthreshold and full attention-deficit hyperactivity disorder in women: a population-based epidemiological study

PLOS One

Dear Dr. Chen

Dear Reviewers

We are grateful for your suggestions and for your careful reading and supportive remarks. Your suggestions helped us to supplement additional technical and substantial information for the readers and to further improve the text.

Please find below our responses to your suggestions and remarks. The responses are coloured in blue.

Kind regards - Vladeta Ajdacic-Gross

Editor's comments

Journal Requirements:

When submitting your revision, we need you to address these additional requirements.

1. Please ensure that your manuscript meets PLOS ONE's style requirements, including those for file naming. The PLOS ONE style templates can be found at

https://journals.plos.org/plosone/s/file?id=wjVg/PLOSOne_formatting_sample_main_body.pdf and

https://journals.plos.org/plosone/s/file?id=ba62/PLOSOne_formatting_sample_title_authors_affiliations.pdf

Hopefully the titles and names are correct.

2. Thank you for stating the following financial disclosure:

“The CoLaus|PsyCoLaus study (represented by MP) is supported by research grants from GlaxoSmithKline (www.gsk.com), the Faculty of Biology and Medicine of the University of Lausanne (https://www.unil.ch/fbm/en/home/menuinst/faculte.html), and the Swiss National Science Foundation (www.snf.ch) (grants 3200B0–105993, 3200B0-118308, 33CSCO-122661, 33CS30-139468, 33CS30-148401, 33CS30_177535 and 3247730_204523) and the Swiss Personalized Health Network (sphn.ch) (grant 2018DRI01).”

Please state what role the funders took in the study. If the funders had no role, please state: "The funders had no role in study design, data collection and analysis, decision to publish, or preparation of the manuscript."

Please include this amended Role of Funder statement in your cover letter; we will change the online submission form on your behalf.

Done.

3. Thank you for stating the following in your Competing Interests section:

“None.”

Please complete your Competing Interests on the online submission form to state any Competing Interests. If you have no competing interests, please state "The authors have declared that no competing interests exist.", as detailed online in our guide for authors at http://journals.plos.org/plosone/s/submit-now

This information should be included in your cover letter; we will change the online submission form on your behalf.

Done.

4. We note that you have indicated that there are restrictions to data sharing for this study. PLOS only allows data to be available upon request if there are legal or ethical restrictions on sharing data publicly. For more information on unacceptable data access restrictions, please see http://journals.plos.org/plosone/s/data-availability#loc-unacceptable-data-access-restrictions.

Before we proceed with your manuscript, please address the following prompts:

a) If there are ethical or legal restrictions on sharing a de-identified data set, please explain them in detail (e.g., data contain potentially identifying or sensitive patient information, data are owned by a third-party organization, etc.) and who has imposed them (e.g., a Research Ethics Committee or Institutional Review Board, etc.). Please also provide contact information for a data access committee, ethics committee, or other institutional body to which data requests may be sent.

b) If there are no restrictions, please upload the minimal anonymized data set necessary to replicate your study findings to a stable, public repository and provide us with the relevant URLs, DOIs, or accession numbers. For a list of recommended repositories, please see

https://journals.plos.org/plosone/s/recommended-repositories. You also have the option of uploading the data as Supporting Information files, but we would recommend depositing data directly to a data repository if possible.

We will update your Data Availability statement on your behalf to reflect the information you provide.

We reformulated the Data Availability statement and introduced the text in the Cover Letter as requested by the Editor:

"Individual-level data cannot be publicly shared by the authors because they contain sensitive, potentially identifiable personal information, and public data sharing was not included in the in-formed consent provided by participants at the start of the CoLaus|PsyCoLaus study (approval by the Ethics Committee of the Canton of Vaud for human research (www.cer-vd.ch; project num-ber PB_2018-00038, reference 239/09)). All relevant aggregated data are available within the paper and its Supporting Information files, where examples of low-level aggregated data are also provided. However, individual-level data from the CoLaus|PsyCoLaus cohort may be accessed by qualified researchers upon reasonable request and following submission of a research application to the research committee of Colaus|PsyCoLaus (see https://www.colaus-psycolaus.ch/professionals/how-to-collaborate), which evaluates requests and grants access in accordance with study regulations."

5. We note that you have included the phrase “data not shown” in your manuscript. Unfortunately, this does not meet our data sharing requirements. PLOS does not permit references to inaccessible data. We require that authors provide all relevant data within the paper, Supporting Information files, or in an acceptable, public repository. Please add a citation to support this phrase or upload the data that corresponds with these findings to a stable repository (such as Figshare or Dryad) and provide and URLs, DOIs, or accession numbers that may be used to access these data. Or, if the data are not a core part of the research being presented in your study, we ask that you remove the phrase that refers to these data.

The sentence comprising the "data not shown" statement was removed.

6. If the reviewer comments include a recommendation to cite specific previously published works, please review and evaluate these publications to determine whether they are relevant and should be cited. There is no requirement to cite these works unless the editor has indicated otherwise.

No recommendations for further publications have been suggested.

Reviewers' comments:

Reviewer's Responses to Questions

Comments to the Author

**Reviewer #1:** I have read the above manuscript and give hereafter my comments:

This study covers an interesting issue, yet several aspects are successively described with a neither convincing nor very clear frame.

1. The major concern would be the confounding factors including the psychiatry comorbidities as well as medications seemed not take into consideration of the analyses, especially when analyzed the risk factors of persistent ADHD.

Thank you for the overall assessment and the constructive specific suggestions.

The reviewer raised two main issues: 1) comorbidities and 2) medication.

1. Comorbidities

While the present manuscript primarily focuses on early-life risk factors and associated features, we also examined comorbidity patterns. There are two reasons why these results were not included here. First, we were concerned that adding further analyses — including ADHD in men or detailed comorbidity results — would substantially increase the complexity of an already demanding manuscript. The current version already requires differentiation between full versus subthreshold ADHD and between occurrence versus persistence.

Second, our publication strategy follows a stepwise approach in which the investigation of ADHD heterogeneity through risk factors represents the foundation. In the present paper, we therefore concentrate on full versus subthreshold ADHD in women and on occurrence versus persistence. A complementary manuscript addressing ADHD in men, as well as manuscripts covering comparative analyses involving tics/Tourette syndrome and ongoing analyses of stuttering, are in preparation. Together, these studies will provide a broader framework for discussing comorbidity patterns across neurodevelopmental disorders and related conditions. A comprehensive comparative background – including differences in age at symptom onset – is helpful to interpret such patterns appropriately.

To provide at least minimal context on this issue, we have added the following statement to point 1 of the Limitations section:"Important perspectives on ADHD heterogeneity are not addressed in the present report – including ADHD in men and comorbidities with other neurodevelopmental and mental disorders – to avoid an overload of information. These aspects will be addressed in subsequent work. "

2. Medication

Medication plays a comparatively minor role in this cohort. The CoLaus|PsyCoLaus study began in the early 2000s, and given the age range of the participants (see Methods), most individuals were born around the mid-20th century (median birth year 1952/1953 in men and women). Pharmacological treatment for ADHD was therefore not available to the majority of participants during their developmental years, young and middle adulthood.

**Reviewer #2:** This study included a broad range of factors, including family psychiatric comorbidity factors, parental bonding, childhood maltreatment, physical health indicators into analysis to differentiate women with subthreshold and full ADHD. As the authors described, some of these factors were defined as the risk factors and some as “associated” factors. I did not see the hypothesis accounting for the analysis.

The reviewer correctly notes that explicit hypotheses are not stated in the manuscript. This reflects the exploratory nature of the study, which we describe in the text. From a methodological perspective, exploratory designs are typically hypothesis-generating rather than hypothesis-testing.

Conceptually, this study resembles exposome wide association studies (EWAS or ExWAS– a twin label to GWAS). Such approaches have become feasible through large epidemiological cohorts – including CoLaus|PsyCoLaus, the UK Biobank, and related initiatives – that provide extensive data on environmental exposures, risk factors, and physiological markers. At the same time, our analytic strategy differs from conventional xWAS frameworks. Rather than isolating single associations or testing specific causal pathways, we focus on patterns across selected variables.

Heterogeneity in ADHD – as in many neurodevelopmental and other complex disorders – is a major obstacle, but it is difficult to address using exclusively hypothesis-driven or strictly reductionist approaches. Exploratory and pattern-oriented methods can therefore complement or replace established methodologies, particularly when heterogeneity emerges from contexts shaped by multisystem complexity. The present study illustrates one possible exploratory strategy within this broader perspective.

The factors included in our analyses were selected because they are likely to occur early in life or to reflect immune and cardiometabolic processes that begin at young ages. We intentionally use the wording “risk and associated factors” to remain cautious with respect to causal interpretation.

Further, these were a small number of women having subthreshold or full ADHD. This is far too different from the real-world situation.

We addressed the potential underreporting of ADHD symptoms and its implications in item 4 of the Limitations section. We would like to clarify that the CoLaus|PsyCoLaus participants belong predominantly to generations born around the mid-20th century, with a median birth year of 1952 and 1953 in men and women. While ADHD incidence and prevalence have changed over time, it remains difficult to determine which real-world situation might have applied to these generations.

A related point may appear counterintuitive at first glance: many higher-level patterns can still be identified in observational data despite challenges such as underreporting, limited sample sizes, or measurement imprecision. This has been demonstrated not only in time-series and longitudinal research but also in cross-sectional analyses. Important caveats arise when strong systematic bias distorts the underlying structure of the data or when relevant patterns are insufficiently represented in the selected variables — one reason why we adopted an exposome-wide perspective. Naturally, interpretability becomes more limited when analyses focus on highly specific patterns or fine-grained details.

**Reviewer #3**: This study investigated risk factors associated with different subgroups of ADHD in women ADHD, using data from a large Swiss population-based epidemiological cohort. The authors examined a broad range of psychosocial (e.g. adverse childhood experiences, parental bonding), as well as biological and physiological indicators (e.g. childhood infections, cardiometabolic and immunological markers). Overall, this is a data-rich study that addresses an important gap in the ADHD literature, particularly with respect to women and ADHD heterogeneity. The strengths of this manuscript are the large, well-characterized population-based sample, its good representativeness and the richness and breadth of indicators considered. Although some indices are necessarily limited to early-life experiences, the overall multidimensional coverage is impressive. The use of latent class analysis to identify subgroups within subthreshold and full ADHD is a notable contribution, highlighting the substantial heterogeneity of ADHD presentations among women. Overall, I consider this manuscript to be important and novel. My comments below are minor and intended to improve clarity and interpretability.

We are grateful to the reviewer for the careful reading and for highlighting the central features of this study.

My primary concern is that the study is highly exploratory and data-driven. This is understandable given the analyses rely on a large, pre-existing epidemiological cohort with a wide array of available measures.

Thank you for this perspective. Large cohort studies such as CoLaus|PsyCoLaus or the UK Biobank — which integrate extensive self-reported data, physiological and somatic measures, imaging, genetic, and omics information — provide opportunities to rethink methodological approaches in risk factor research. The present study aims to illustrate a systematic exploratory analysis framework in conditions shaped by complexity and heterogeneity. Conceptually, it draws on exposome-wide association approaches (ExWAS, analogous to GWAS), but takes a different direction than conventional xWAS strategies. Rather than isolating individual associations in detail, we focus on patterns emerging from relationships among selected variables. In addition, this framework is combined with a comparative analytic perspective, as exemplified in the present study.

However, the manuscript would benefit from a more explicit integrative model of heuristic framework. In particular, the authors could strengthen the discussion by addressing how their findings might translate into practical implication, such as early identification of women at higher risk for persistent ADHD, stratification of ADHD subtypes for prognosis, or potential targets for early intervention.

Thank you for emphasizing this important point — we very much share the reviewer’s perspective. At the same time, we are faced with the challenge that the present work is an exploratory, association-based study, which requires cautious interpretation. Although approaches such as GWAS or machine- and deep-learning frameworks have demonstrated the value of exploratory analyses, such work is sometimes met with skepticism, particularly when the methodological rationale is not fully appreciated. We therefore aimed to balance openness to emerging patterns with careful and conservative interpretation.

At the same time, this study offers a concise illustration of what may be expected from future research in this area. We agree that, in ADHD as in other disorders, mechanisms underlying persistence may represent important opportunities for intervention. Distinguishing processes that occur before and after the biological onset of a complex disorder is likely to be essential, with potentially far-reaching implications.

Following the reviewer’s suggestion, we have added the following text to the summary paragraph of the Discussion on " Risk and associated factors for ADHD persistence in women":

"Persistence has emerged as a topic that is no less important for understanding ADHD outcomes than the occurrence of ADHD itself.

...

These factors and processes introduce a challenging new perspective, as all of them represent potential targets for intervention. Moreover, disentangling the heterogeneity of ADHD may provide important clues for applying interventions in a subgroup-specific manner. Research in this direction is ongoing {Chang, 2018 #693} and may further benefit from parallel efforts in studies of other disorders {Worrell, 2025 #694;Yang, 2025 #695;Palmer, 2025 #696}."

In addition, we removed the following sentence from this paragraph:
"Regarding the latter, there are conspicuous differences between full and subthreshold ADHD, which might be related either to the different severity or to the different age of onset."

Minor comments

1. The selection of markers for LCA/LPA was based on a significance threshold p<0.1 for associations with ADHD. The rationale for this decision is unclear and should be more explicitly justified.

We appreciate the reviewer’s interest in the methodological details of the LCA procedure. In an LCA framework, two main challenges arise when selecting variables. On the one hand, we aim to apply less restrictive inclusion criteria than the conventional thresholds (e.g., p < .05) typically used. This reflects the heterogeneity of targets such as ADHD: experience from less restricted analyses of bigger samples shows that small classes may be characterized by variables whose initial associations display relatively large p-values (sometimes .5 or higher). On the other hand, including too many variables can also be problematic, as LCA solutions are highly sensitive to the configuration of variables, for example when additional system dimensions are introduced.

To address this dilemma, we developed and applied a stepwise alternative approach. First, we conducted an initial LCA using relatively restrictive inclusion criteria (here p < .1, given the low cell frequencies). Second, additional variables associated with the initial classes were incorporated based on the same inclusion threshold. Optionally, the LCA can be repeated following this second step, and the procedure can be iterated further if required. This strategy also has limitations: while the aim is to identify configurations that differentiate ADHD-related patterns from those typical of the general population, repeated iterations may gradually shift the solution toward more general population configurations. In the present analysis, we therefore stopped after the first iteration, also considering the limited frequencies available.

Because the discussion of LCA methodology was already extensive in the manuscript, we initially chose not to include this additional level of detail. However, following the reviewer’s suggestion and to improve clarity, we added the following text:

" The focus is typically placed on variables that show a significant or trend-level association with ADHD, although this represents an imperfect criterion. Heterogeneity implies that associations of variables belonging to smaller classes may be suppressed, and these variables can therefore be overlooked during the selection procedure. Two basic strategies can be used to address this dilemma. The first borrows from a common variable-selection approach in regression analysis by setting a relatively lenient cutoff, for example p < 0.25, after initial screening{Hosmer, 2000 #687}.

...

The second strategy, applied here, is to begin the selection of variables markers was based on the a less lenient significance level, ... ".

2. Table 2. It is unclear why for the same indicator, the total n differs between the subthreshold ADHD group and the full group.

Thank you for this precise remark. The total n in analyses with full ADHD did not comprise participants with subthreshold ADHD (and vice versa). We added a note in Tables ...

3. When reporting statistics, it would be preferable to report the relevant test statistics (e.g. t value and degrees of freedom), not only the p value (e.g. Line 448), to improve transparency.

The instances addressed by the reviewer relate mainly to the reporting of the age of onset, which was in the format "mean(standard error)". We now changed the format to the one used in reporting of the physiological markers. To keep it consistent across dichotomous and polytomous variables we report F-values.

4. Line 515 Please check whether the “ACE high BP” group corresponds to Class 3

Thank you for this helpful suggestion. Upon rechecking the data, we found that an error had occurred during the updating of the text referring to Table 3c. We have revised the corresponding paragraph and inserted the corrected values.

5. Line 762 The discussion that “lower blood pressure may reflect a distinct subtype of subthreshold ADHD” is intriguing. The authors might consider whether this pattern could relate to concepts such as Cognitive Disengagement Syndrome (or sluggish Cognitive Tempo), autonomic hypoarousal, or alternative neurobiological profiles.

This is an interesting idea, which we followed up by examining associations with symptoms characteristic of the inattentive subtype of ADHD. However, these additional analyses did not yield meaningful or interpretable results.
