# Supplementary material for: Factors associated with the occurrence and persistence of subthreshold and full attention-deficit hyperactivity disorder in women: A population-based epidemiological study
Source: PLoS One. 2026 May 14;21(5):e0340179. doi: 10.1371/journal.pone.0340179 (PMC13175469; doi:10.1371/journal.pone.0340179)
Supplement: Attachment — Submitted filename: Response to Reviewers PONE-D-25-65436Revision2.docx [file pone.0340179.s003.docx]

PONE-D-25-65436R1

Factors associated with the occurrence and persistence of subthreshold and full attention-deficit hyperactivity disorder in women: a population-based epidemiological study

PLOS One

Dear Dr. Chen

Dear Reviewer

Thank you for your follow-up regarding confounding factors in our manuscript. We appreciate the opportunity to continue this discussion and to provide additional context on ADHD in Switzerland.

Please find below our responses to your suggestions and comments. Responses are highlighted in blue.

Kind regards - Vladeta Ajdacic-Gross

**Additional Editor Comments / Reviewer 1:**

The present comments refer to Comment 1 of the Reviewer on the first version of the manuscript:

"The major concern would be the confounding factors including the psychiatry comorbidities as well as medications seemed not take into consideration of the analyses, especially when analyzed the risk factors of persistent ADHD."

This concern is reflected in the present comment 2 of the Reviewer. Below, we address Part 1:

"The authors partially replied to my comment about the confounding factors, including comorbidities and medications. They plan to conduct further analysis about the comorbidities in the future."

We appreciate the reviewer’s understanding of our research and publication strategy. With regard to psychiatric comorbidities, we acknowledge that these were not systematically included in the present analyses. As noted in the first revision, we have explicitly addressed this as a limitation in the revised manuscript (Methods and Discussion sections) and have outlined plans for future analyses focusing on comorbidity profiles. To date, our analyses have focused on comorbidities involving infectious and other somatic diseases, which typically occur or begin early in life and may act as potential risk factors.

It is important to note that the analysis and interpretation of comorbidities represent one of the most challenging aspects of research on neurodevelopmental and mental disorders, despite appearing straightforward at first glance. These conditions are inherently complex and heterogeneous, and ADHD is no exception.

Analyzing comorbidities involves, among other factors, the intersection of multiple layers of heterogeneity. Consequently, preliminary methodological work is required, for example through network analysis or – as pursued in the present study – through a combined comparative and pattern recognition approach.

The following addresses Part 2 of the reviewer’s comment:

"The authors also argued that medication plays a comparatively minor role in this cohort because pharmacological treatment for ADHD was not available to the majority of participants during their developmental years, young and middle adulthood (median birth year 1952/1953). However, data shows that the FDA has approved Methylphenidate (MPH) for ADHD treatment as early as early 1960s. Therefore the concern about confounding factors still existed."

We agree that the availability of methylphenidate should be considered when evaluating potential confounding by medication. This issue can be viewed from both an empirical and a historical perspective.

Empirical perspective:

The CoLaus|PsyCoLaus cohort study dataset includes information on the use of several medications that may be prescribed for ADHD, as well as for other disorders, including methylphenidate (Ritalin), bupropion (Wellbutrin), lisdexamfetamine (Elvanse), and atomoxetine (Strattera), as well as a category labeled “other stimulants.” Among the 11 women in the CoLaus|PsyCoLaus cohort who reported, either at baseline or during follow-up assessments, having used any of these medications, one woman met criteria for full ADHD, and none met criteria for subthreshold ADHD. As these data refer to lifetime medication use, they also capture aspects of the historical context of treatment exposure.

Historical perspective:

Methylphenidate was introduced in Europe in the 1950s and was initially used for a broad range of non-specific indications (e.g., fatigue, behavioral difficulties), in some contexts even with limited regulatory restrictions. At that time, however, no standardized diagnostic framework for ADHD existed. Early classifications such as “hyperkinetic reaction of childhood” (DSM-II, 1968) and “hyperkinetic syndrome of childhood” (ICD-9, 1975) were narrowly defined, inconsistently applied, and restricted to pediatric populations.

Importantly, ADHD was not recognized as a disorder persisting into adulthood until much later, with broader clinical acceptance emerging only from the 1990s onwards (DSM-IV/ICD-10 era). Consequently, systematic diagnosis – and therefore treatment – during adolescence and adulthood was largely unavailable for this cohort (median birth year 1952/1953).

In Switzerland, the closest related construct at the time was the so-called psycho-organic syndrome (POS), introduced in 1971 as part of the disability insurance framework. Importantly, POS was not a standardized clinical diagnostic category comparable to modern ICD/DSM definitions, but rather an administrative construct with restrictive criteria, and was not broadly implemented in routine psychiatric care.

Taken together, these factors indicate that although methylphenidate was pharmacologically available, its use was unlikely to have been systematic, diagnosis-driven, or widely implemented in this population. As pharmacological treatment presupposes a prior diagnosis, overall exposure to stimulant medication in this cohort is expected to have been low and non-systematically distributed.

In summary, we consider it unlikely that medication exposure constitutes a major source of bias in the present analyses. While regulatory approval or early market availability may suggest availability, this does not necessarily translate into systematic, diagnosis-driven clinical use, particularly across different healthcare systems and historical periods. Nevertheless, we acknowledge that residual confounding cannot be entirely excluded, for example due to underreporting, and we have clarified this limitation in the revised manuscript.

We have supplemented the text as follows (line 841):

"Fourth, information related to symptoms, comorbid diseases / disorders and medications used for therapy was obtained retrospectively and may be subject to recall bias."
